# The Color “Fruit”: Object Memories Defined by Color

**DOI:** 10.1371/journal.pone.0064960

**Published:** 2013-05-22

**Authors:** David E. Lewis, Joel Pearson, Sieu K. Khuu

**Affiliations:** 1 The School of Psychology, The University of New South Wales, Sydney, Australia; 2 The School of Optometry and Vision Science, The University of New South Wales, Sydney, Australia; University of California, San Francisco, United States of America

## Abstract

Most fruits and other highly color-diagnostic objects have color as a central aspect of their identity, which can facilitate detection and visual recognition. It has been theorized that there may be a large amount of overlap between the neural representations of these objects and processing involved in color perception. In accordance with this theory we sought to determine if the recognition of highly color diagnostic fruit objects could be facilitated by the visual presentation of their known color associates. In two experiments we show that color associate priming is possible, but contingent upon multiple factors. Color priming was found to be maximally effective for the most highly color diagnostic fruits, when low spatial-frequency information was present in the image, and when determination of the object's specific identity, not merely its category, was required. These data illustrate the importance of color for determining the identity of certain objects, and support the theory that object knowledge involves sensory specific systems.

## Introduction

Color can be highly informative of an object's identity. Accordingly, most animals have some ability to perceive color, including mammals which typically have dichromatic color vision [1], [2]. The trichromatic color vision of humans and other primates is a relatively new genetic development, allowing for improved discrimination between various shades of red and green [3]. This evolution is theorized to have been driven by the color of tropical fruits [4]–[6], resulting in the development of a visual system especially well tuned to long wavelength colors (red, orange and yellow), which often signal the most nutritious fruit [7]. These evolutionary theories of color vision have gained popularity and support in recent years, but typically make no attempt to explain contributions of higher-level cognitive processes, such as how an object's color information is encoded, organized and recalled from memory.

Many objects, such as ripened fruit, tend to have specific colors. For example, ripe bananas and lemons are always colored yellow. Such objects are commonly referred to as being highly color diagnostic, as their colors will provide additional information about their identity [8]. As a result when the shape of an object is made ambiguous through image blur, its colors can have a strong facilitatory effect on its recognition [9]. This improved ability to identify an object may simply involve using color channels to help define its contours, as suggested by numerous studies [10]–[12]. However, studies using highly color-diagnostic objects have found that the addition of color information leads to greater facilitation for these objects when compared to those less linked to color [13]–[16]. This color based facilitation appears to result in the activation of a more extensive neural network than colorless images, which makes object recognition faster [17]. These findings illustrate how object recognition can be facilitated by the color specific information stored within memory.

Memory of these object-color associations can also greatly influence the apparent coloration of a visually presented object. A seminal study on such memory color effects [18] has demonstrated that an object's perceived color is skewed towards what the observer expects it to be, based on knowledge gained from previous experience. These memory colors are independent of the influence of color constancy in that they are observable even when all objects in a scene are uniformly colored or when an object is presented in isolation. Therefore, while color constancy attempts to determine the “true” color of an object by estimating and then discounting the color of the illumination [19], the memory color effect adjusts the perceived colors of an object to match the association held within memory. This effect occurs involuntarily and has been demonstrated for both natural objects [20]–[23] and man-made objects [24]. Along with the previously discussed literature, this memory color effect shows that an object's identity and its coloration can be highly interactive. However, the mechanisms underlying these interactions remain largely unknown.

There appears to be an overlap of the neural areas involved in the processing of color perception and stored color knowledge [25]–[27]. Such an overlap would identify the mechanisms of object identity-color interactions and support the theory that conceptualized knowledge is grounded within modality specific systems [28]. According to this theory the storage and retrieval of knowledge about an object, such as its color, involves processing within the same neural areas involved during the perception of that object.

However, the functional independence of color perception and color knowledge has been demonstrated in multiple case studies [29]–[31], and implicated in many neuroimaging studies [17], [32]–[34]. These findings indicate it is possible to recall color knowledge from memory without being able to process perceptual colors, and vice-versa. If these two processes can truly operate independently of one another it would be indicative of a double dissociation of color perception and color knowledge. These results conflict with the theory of modality grounded knowledge, leading to a great deal of debate [28]. If perceptual color processing were clearly shown to have an effect on the retrieval of object color knowledge this would support the theory of modality grounded knowledge as applied to color perception, which would in turn facilitate a resolution to this ongoing debate.

Many behavioral studies have already made important contributions to this resolution. It has been shown that object recognition can be interfered with through the prior presentation of the name or picture of an object known to be the same color [35], [36]. This appears to be a kind of color-knowledge-based negative priming that will only occur if the observer knows that the two objects are typically the same color. However, this interference has been shown to only occur when the object to be recognized is shown in its appropriate color [37]; no recognition interference was observed for colorless or inappropriately colored objects. This indicates that color knowledge can play a role in object recognition processing, but it appears as though this knowledge may require activation by the visual coloration of the object to be recognized [38].

In contrast, a type of knowledge-based positive color priming has recently been demonstrated in a study showing that the names of objects known to share similar colors can prime each other in a word recognition task [39]. As these object names were merely colorless words, this priming did not require the presentation of any visual colors. This form of priming is based on the object's known color associates, indicating that the stored knowledge of similarly colored objects appear to have overlapping neural representations. However, this priming was entirely semantic and did not involve the processing of any perceptual colors, therefore it does not demonstrate that object knowledge requires perceptual color processing. It may simply be that there is an overlap between the neural representations of these objects that does not involve any perceptual processing.

The previously mentioned studies have shown that color knowledge can play an important role in visual object recognition. However, whether sensory colors can have an influence on stored color knowledge has not yet been clearly demonstrated. The demonstration of this kind of influence would suggest that processing for color perception and color knowledge involve the same neural mechanisms, as predicted by the theory of modality grounded cognition. The aim of the present study is to build upon these previous findings by showing that such an interaction is possible.

In the current study we sought to determine if perceptual colors can interact with the stored knowledge of highly color diagnostic objects. Specifically, we tested whether the recognition of achromatically presented fruit objects can be facilitated using visual color primes. Achromatic presentation of the fruit objects ensures that the only possible links between them and the perceptual color primes are those stored within memory. If significant facilitation is found using such a priming task it would suggest that memory for highly color diagnostic objects at least partially involves similar neural processing as their perceptual colors. Such a result would provide compelling support for the theory that object color knowledge is grounded in perceptual processing areas [28].

We assessed the effectiveness of our color primes using two separate experimental tasks, object naming (Experiment 1) and object categorization (Experiment 2). As the presence of an object's surface details are known to have different effects on its categorization and naming [40], it seems possible that our color primes would also have different effects in these two tasks. In the categorization task observers were merely required to categorize the objects as either a “Fruit” or “Car”, they were not required to determine the objects' specific identities. As a result, our color primes may be ineffective in this task, as it is the specific identities of the fruit that are known to be associated with different colors. This result seems especially likely as visual objects are known to be categorized before their specific identities can be determined [41]–[43].

In both experiments we tested this priming under different visibility conditions. Previous object recognition studies have shown that visual colors can produce greater facilitation for blurred or degraded object images [16], [40], [44], suggesting that our color priming may also be maximally effective under these conditions. Therefore, in addition to normal achromatic photo-images, we tested highly blurred images that preserved the low spatial-frequency luminance information while removing most of the objects' shape and texture. In Experiment 1 we also tested an edges-only version of the photographs that preserved the objects' high spatial-frequency shape and texture while removing most of the luminance information. To reiterate, there were no shared visual features between our color primes and the achromatic object targets used in any of these experiments. Therefore, any observed priming must be based upon on object specific color knowledge stored in memory [26].

## Materials and Methods

### Ethics Statement

This study received ethical approval from the University of New South Wales Human Research Ethics Advisory (HREA) panel. All participants gave written, informed consent before the start of the experiment. Upon completion the participants were debriefed in a follow-up interview.

### Participants

A total of 21 students (9 males) were recruited from the University of New South Wales for this study (*n_naming_* = 11; *n_categorization_* = 15). All participants had normal, or corrected to visual acuity and color vision. Five individuals participated in both the naming and categorization experiments in separate sessions.

### Stimuli

All stimuli were presented using MATLAB (version 7.10.0 R2010a). Experiment 1 used a 32 cm×51 cm iMac monitor with a Nvidia GeForce GT 120 chipset at a resolution of 1920×1200. Experiment 2 used a 27 cm×35.5 cm Philips 109P4 monitor at a resolution of 1152×870 @ 75 Hz. Both experiments involved the presentation of colored Gaussian blobs and achromatic photographs on a black background (luminance <0.01 cd/m^2^). Stimuli were presented in the center of the screen with a circular fixation mark overlaid (diameter = 0.3°).

The Gaussian blobs were created using Psychtoolbox [45] for MATLAB. Six differently colored Gaussians (σ_width_ = 2.7°; σ_height_ = 1.8°) were used in this study: red (CIE x = 0.628 y = 0.338 lum = 61.7 cd/m^2^), orange (CIE x = 0.506 y = 0.431 lum = 110 cd/m^2^), yellow (CIE x = 0.392 y = 0.515 lum = 234 cd/m^2^), yellow-green (CIE x = 0.344 y = 0.553 lum = 134 cd/m^2^), green (CIE x = 0.279 y = 0.600 lum = 200 cd/m^2^) and blue (CIE x = 0.142 y = 0.071 lum = 24 cd/m^2^). These colors were chosen due to their relevance to fruit perception, except blue which acted as a control. These colors were not isoluminant.

**Figure 1:**
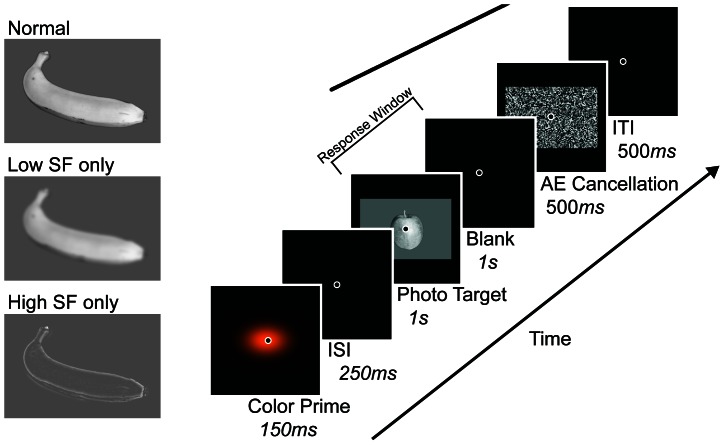
Example photographic stimuli and experimental timeline. (*Left*) Example photograph targets as shown in each of the three conditions. All photographs were presented on a black screen, in grayscale, with their backgrounds removed. (*Right*) Experimental timeline. In Experiment 2 the blank screen immediately following the photograph target was removed, thus shortening the response window to only 1 s.

The photographs chosen for this study consisted of various kinds of fruit and cars. Fruit photographs were taken in the lab using a Nikon D50 camera and store-bought fruits. All fruits were photographed from a canonical angle; as though the viewer is looking down at them upon a table. Any photographs containing extraneous or distracting visual features such as blemishes, discolorations, and lighting effects were discarded. The chosen fruit types were apples, bananas, grapes, lemons, oranges, pears, persimmons and strawberries. Fruit types were selected based upon their familiarity and color diagnosticity as determined in previous color diagnosticity studies using fruit stimuli [15], [16], [46]. The fruit rated high in color diagnosticity were bananas (yellow), lemons (yellow), oranges (orange), and strawberries (red), while those rated moderate to low in color diagnosticity were apples, grapes, and pears. Persimmons were included as an experiential control, as it is our understanding that they have not been used in any prior color diagnosticity experiments. This is likely because most people have no knowledge of persimmons, and knowledge is required for an object's color to be diagnostic of its identity. Consequently, though persimmons always appear in a particular shade of reddish-orange, none of the participants had this knowledge, making it a non-color diagnostic object. Car photographs were found using various internet sources. The car photographs were included as a non-fruit control condition, as cars have been consistently rated very low in color diagnosticity in previous research. The chosen car types are classic cars, convertibles, luxury cars, sedans, smart cars, sport-utility vehicles, pickup-trucks and vans. All objects were displayed in achromatic grayscale with their backgrounds removed. These objects were centered and resized to fit on top of a gray rectangle (width = 16.2°; height = 10.9°). See the left side of Figure 1 for example stimuli.

In Experiment 1, the naming experiment, a total of 54 photographs were used: six photographs of each kind of fruit, except persimmons, and only one photograph of each kind of car, except sports-cars, pickup-trucks and vans. This resulted in a total of 8 different kinds of objects used in this experiment. Depending on the experimental condition, the photographs were filtered to remove either the low or high spatial-frequency information. The low spatial-frequency versions were created by convolving each photograph with a 2-D Gaussian filter of the same size as the photographs and a standard deviation of 5 pixels (σ≈0.3°). The high spatial-frequency versions were created by subtracting the low spatial-frequency versions from the original photographs.

In Experiment 2, the categorization experiment, a total of 96 photographs were used: six photographs of each kind of fruit and car. Only the normal photographs and low spatial-frequency versions were used in this experiment.

### Design

The two experiments in this study utilized a 6×8 within subjects design. The two independent variables were prime color and object type. In each experiment the potential interactions between the levels of prime color and object type were tested independently for each spatial-frequency condition. In Experiment 1 there were three spatial-frequency conditions (normal, low spatial-frequencies only, high spatial-frequencies only) while in Experiment 2 there were only two spatial-frequency conditions (normal, low spatial-frequencies only).

In Experiment 1 the different object types were apples, bananas, grapes, lemons, oranges, pears, strawberries and cars. In Experiment 2 the potential interactions between prime color and object type were independently tested for each of the two object categories (fruit, car). This independent analysis was conducted to more directly compare the effects of the six differently colored primes across the eight object types within each category. The fruit types were apples, bananas, grapes, lemons, oranges, pears, persimmons and strawberries. The car types were classic cars, convertibles, luxury cars, sedans, smart cars, sport-utility vehicles, pickup-trucks and vans. No cross-category comparisons were made in this categorization experiment.

### Procedure

All participants were seated in a dark room approximately 57 cm from the monitor. A chinrest was not used in Experiment 1 as it would interfere with the participants' ability to vocalize their responses. The basic experimental timeline shown on the right side of Figure 1 was used in both experiments. At the start of each trial the Gaussian color prime was shown for 150 ms, followed by a 250 ms blank screen. Then the target photograph was shown for 1 s, followed by a 1 s blank screen. Participants were only allowed to vocalize their responses during this 2 s response window. Then a randomized white noise pattern was displayed for 500 ms to remove any luminance after-effects produced by the photographs, which was followed by a 500 ms inter-trial interval wherein the screen would remain blank.

In Experiment 2 the blank screen immediately following the target photograph was removed, thus shortening the response window to only 1 s. This shortening of the response window was due to the observation that the button-press reaction times were generally much quicker than the vocalized response reaction times. Failure to react within the response window for either experiment resulted in the trial being repeated at a randomly determined point later in the session.

In Experiment 1 the participants were instructed to speak aloud the name of the object being displayed in the target photograph as quickly and accurately as possible. Participants were urged to vocalize whichever term they best associated with the object's specific identity (i.e., “apple”). The only exception to this was the car photographs, which were responded to simply as “car”.

In Experiment 2 the participants were instructed to categorize the objects in the target photographs as a fruit or car by pressing the “F” or “C” keys on the keyboard as quickly and accurately as possible. Incorrect responses resulted in the trial being repeated at a randomly determined point later on in the experimental session and a buzzer noise was played to notify the participants of their error.

### Data preparation

In Experiment 1 the naming accuracy and vocalization reaction time for each trial were manually determined after the experimental session was completed. The visual waveform and auditory playback were reviewed for each trial using a MATLAB program written specifically to analyze the sound-waves of this study. Reaction times were determined by marking the point of vocalization onset, as indicated by systematic changes in the waveforms' amplitudes while ignoring ambient noises and non-verbal utterances. Variations in the waveform shapes due to individual participant differences were also taken into consideration when marking this onset of vocalization. Accuracy was determined by comparing the auditory playback to the correct answer being textually displayed in the program window. The raters were blind as to the color prime of each trial; only the participant's vocalization and the correct answer were shown.

In Experiment 2 the program automatically determined the accuracy and reaction time of each participant's button-press responses. All incorrect responses were recorded and the trial was repeated at a randomly determined point later in the block. Incorrect responses were excluded from analysis in both experiments due to the participants having made very few errors. In the normal-image condition of Experiment 2 a single participant was excluded from analysis due to consistently producing outlying reaction times.

## Results

### Experiment 1 - Naming

In this experiment we sought to determine if naming speeds for the achromatic fruit images could be facilitated through the presentation of color primes. The priming was tested using photographs that contained different kinds of spatial-frequency information (normal photographs, low spatial-frequencies only, high spatial-frequencies only). Our hypothesis predicted an interaction between the color primes and the object types, with diagnostic colors resulting in the fastest reaction times. It was also predicted that the priming effects would be strongest in the low spatial-frequency condition, as it has been shown that an object's color will maximally facilitate its recognition when the shape is hard to determine [16], [40], [44].

A preliminary 3-way within-subjects ANOVA across the 3 spatial-frequencies, 6 prime colors, and 8 object types was conducted. A significant interaction was found between these three factors *F* (70, 700) = 1.51, *p* = 0.006, observed 1−β = 1.00. This indicates that the removal of certain kinds of spatial-frequency information had a significant effect on the color primes' ability to facilitate the recognition for the target objects. However, this result does not indicate whether a statistically significant amount of color-associate priming was found in any or all of the spatial-frequency conditions. Additional tests were conducted to more directly address the main hypothesis.

**Figure 2:**
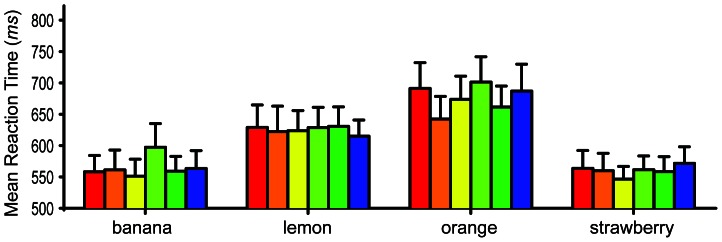
Naming reaction times for highly color-diagnostic objects. Bar color indicates prime color. Error bars show +1 SEM. *n* = 11.

For the normal, unfiltered photographs a 2-way within-subjects ANOVA across the 6 prime colors and the 8 object types was conducted. A significant interaction was found between object type and prime color *F* (35, 350) = 1.50, *p* = 0.039, observed 1−β = .991. As can be seen in Figure 2, the color orange was the most effective color prime for oranges, but the diagnostic colors of the other fruits do not seem to be any more effective than the non-diagnostic colors. A total of 20 Bonferroni corrected analyses were conducted for the highly color-diagnostic objects to determine if their diagnostic colors resulted in significantly faster naming speeds than their non-diagnostic colors. For the bananas, yellow was found to produce significantly faster naming speeds than yellow-green (*t* = 3.61, *p*<0.001). For the oranges, the color orange was found to produce significantly faster naming speeds than red (*t* = 3.82, *p*<0.001), yellow-green (*t* = 4.61, *p*<0.001), and blue (*t* = 3.49, *p*<0.001). For the lemons and strawberries no significant differences in naming speed were found. Though only two of the four highly color-diagnostic fruits showed significant differences between their diagnostic and non-diagnostic colors, these findings support our hypothesis by showing significant priming effects driven by the known color associates of the achromatically presented fruits.

A significant main effect of color prime was also found *F* (5, 50) = 3.54, *p* = 0.008, observed 1−β = .887, indicating that one or more of the color primes produced significantly faster reaction times than the others across all object types. Additionally, a highly significant main effect of object type was found *F* (7, 70) = 27.14, *p*<0.001, observed 1−β = 1.00, indicating that some of the objects could be named faster than the others regardless of prime color. This effect can be clearly seen in Figure 2, wherein reaction times for the bananas and strawberries were much faster than lemons and oranges. This illustrates that the distinctiveness of the objects' shapes had a large influence on their recognition speeds.

For the high spatial-frequency photographs a 2-way within-subjects ANOVA across the 6 prime colors and the 8 object types was conducted. No significant interaction was found between object type and prime color *F* (35, 350) = 0.88, *p* = 0.664, observed 1−β = .851. This indicates that the priming was ineffective when the low spatial-frequencies were removed from the object photographs. Only a marginally significant main effect of prime color was found *F* (5, 50) = 2.32, *p* = 0.057, observed 1−β = .697. However, a highly significant main effect of object type was found *F* (7, 70) = 24.41, *p*<0.001, observed 1−β = 1.00, once again indicating that some of the objects could be named faster than others regardless of the color of the prime.

**Figure 3:**
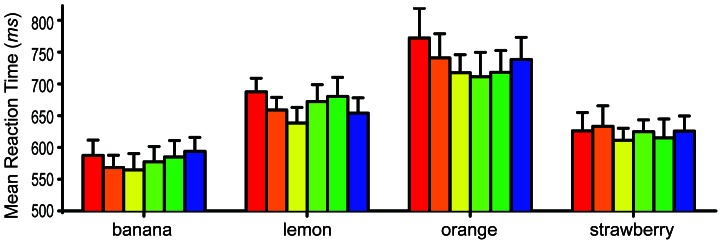
Naming reaction times for the highly color-diagnostic low spatial-frequency objects. Bar color indicates prime color. Error bars show +1 SEM. *n* = 11.

For the low spatial-frequency photographs a 2-way within-subjects ANOVA across the 6 prime colors and the 8 object types was conducted. A significant interaction was found between object type and prime color *F* (35, 350) = 1.55, *p* = 0.027, observed 1−β = .993. As can be seen in Figure 3, the most effective color prime was *yellow* for the bananas and lemons, but *orange* was not the most effective for oranges, and *red* was not the most effective for strawberries. A total of 20 Bonferroni corrected analyses were conducted to determine if there were any differences in the naming speeds for each highly color-diagnostic object's diagnostic color and its non-diagnostic colors. For the lemons, yellow was found to produce significantly faster naming speeds than red (*t* = 3.62, *p*<0.01). For the bananas, oranges, and strawberries no significant differences in naming speed were found. Though only one of the four highly color-diagnostic fruits showed significant differences between their diagnostic and non-diagnostic colors, this finding supports our hypothesis by showing significant priming effects driven by the known color associates of the achromatically presented fruits.

Additionally, for the low spatial-frequency photographs a significant main effect of prime color was found *F* (5, 50) = 4.26, *p* = 0.003, observed 1−β = .942, indicating that the color primes produced significantly different reaction times across all object types. A highly significant main effect of object type was also found *F* (7,70) = 42.54, *p*<0.0001, observed 1−β = 1.00, indicating that the participants were able to name some of the objects quicker than others regardless of prime color.

### Experiment 2 - Categorization

In Experiment 2 we sought to determine if similar results could be produced using a categorization task in place of the naming task. Though we made the same predictions as Experiment 1, it seemed likely that our color primes would have different effects in this categorization task, as an object's color is known to have different effects on its categorization and naming [40]. However, as cars are generally not color diagnostic, we predicted no interaction between the color primes and car types. For various reasons 10 of the 15 individuals who participated in this experiment only completed one of the two conditions (*n*_both_ = 5, *n*_normal_only_ = 7, *n*_LSF_only_ = 3). Though it would have been preferable to have all participants complete both conditions, this is not problematic to the following analyses as they compare differences within the two spatial-frequency conditions, not across them. Note also that while the labels “fruit” and “car” might not be at the same categorical level [47], the car stimuli were merely used as a control condition and no statistical analyses compared these two categories of objects; the data were split by category (“fruit” vs “car”) to facilitate the comparison of the objects within each. Preliminary 3-way within-subjects ANOVAs similar to that of Experiment 1 were not conducted for this experiment as many participants were unable to complete both of the spatial-frequency conditions.

**Figure 4:**
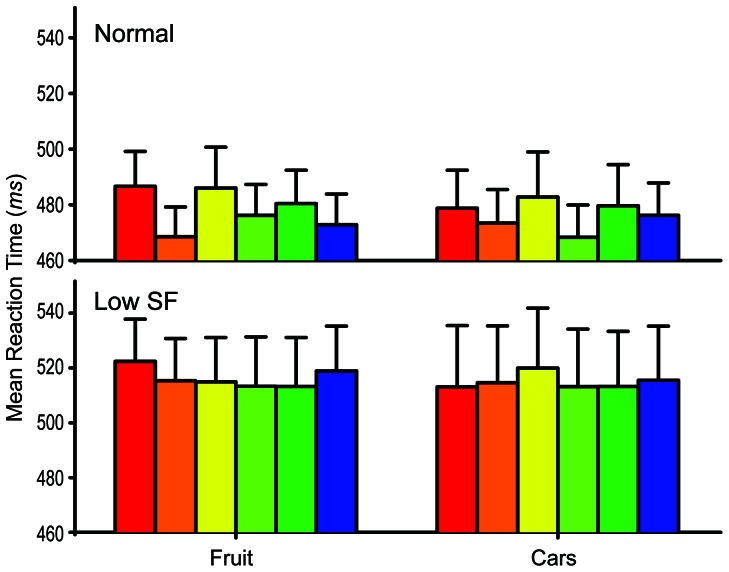
Mean categorization reaction times across all objects separated by category and spatial-frequency condition. (*Top*) Normal: Unfiltered target photographs *n* = 12. (*Bottom*) Low SF: Low spatial-frequency target photographs *n* = 8. Bar color indicates prime color. Error bars show +1 SEM.

For the normal fruit photographs a 2-way within-subjects ANOVA across the 6 prime colors and the 8 fruit types was conducted. No significant interaction between fruit type and prime color was found *F* (35, 385) = 1.32, *p* = 0.108, observed 1−β = .979. Like Experiment 1, a significant difference in the mean reaction times was found between the 8 fruit types *F* (7, 77) = 3.74, *p* = 0.002, observed 1−β = .968. This seems to indicate that some of the fruit objects were easier to recognize as belonging to the “fruit” category. Also, a highly significant difference was found between the 6 prime colors *F* (5, 55) = 4.21, *p* = 0.003, observed 1−β = .941, suggesting that some colors were better than others at priming objects within the “fruit” category. As can be seen on the top-left side of Figure 4 the color orange appeared to produce the fastest reaction times regardless of fruit type. A total of 5 Bonferroni corrected analyses were conducted to determine if the reaction times for orange were significantly faster than the other 5 colors across the 8 different fruit types. The color orange produced faster reaction times than red (*t* = 3.62 *p* = 0.003), and yellow (*t* = 3.50 *p* = 0.0045).

For the normal car photographs a 2-way within-subjects ANOVA across the 6 prime colors and the 8 car types was conducted. No significant interaction between car type and prime color was found *F* (35, 385) = 1.17, *p* = 0.108, observed 1−β = .955. No significant difference in the mean reaction times was found between the 8 car types *F* (7, 77) = 0.87, *p* = 0.534, observed 1−β = .362. This seems to indicate that all of the car objects were equally easy to recognize as belonging to the “car” category. No significant difference was found between the 6 prime colors *F* (5, 55) = 1.66, *p* = 0.160, observed 1−β = .534. However, as can be seen on the top-right side of Figure 4 the color yellow-green appeared to produce the fastest reaction times regardless of car type.

For the low spatial-frequency fruit photographs a 2-way within-subjects ANOVA across the 6 prime colors and the 8 fruit types was conducted. Unlike the normal spatial-frequency condition a significant interaction between fruit type and prime color was found *F* (35, 245) = 1.56, *p* = 0.028, observed 1−β = .992. A total of 20 Bonferroni corrected analyses were conducted to determine if there were any differences in the categorization speeds for each highly color-diagnostic object's diagnostic color and its non-diagnostic colors, but no significant differences were found.

Unlike the normal spatial-frequency condition, no significant difference in the mean reaction times was found between the 8 fruit types *F* (7,49) = 1.24, *p* = 0.302, observed 1−β = .473. This indicates that when high spatial-frequency information is removed from the images, the fruit objects are equally easy to recognize as belonging to the “fruit” category. No significant difference in the mean reaction times was found between the 6 prime colors *F* (5, 35) = 1.79, *p* = 0.14, observed 1−β = .545.

For the low spatial-frequency car photographs a 2-way within-subjects ANOVA across the 6 prime colors and the 8 car types was conducted. No significant interaction between car type and prime color was found *F* (35, 245) = 0.68, *p* = 0.912, observed 1−β = .693. However, a highly significant difference in the mean reaction times was found between the 8 car types *F* (7, 49) = 5.31, *p*<0.001, observed 1−β = .995. No significant difference was found in the mean reaction times between the 6 prime colors *F* (5, 35) = 0.59, *p* = 0.710, observed 1−β = .191.

## Discussion

This study demonstrates the priming of object recognition using known color associates. Facilitating the recognition of achromatic objects through prior exposure to a known color associate provides evidence of a functional relationship between the processing of color perception and color knowledge (eg. memory). This novel finding was observed using fruit stimuli and appears to have been driven by the color diagnosticity of the fruit objects, the low spatial-frequency information of their images, and the level of identity specificity required for the recognition task. To aid in the demonstration of these color priming effects the current study specifically utilized a within-subjects design, small sample-size, and selective range of stimuli. Unfortunately these design choices limit the generality of the current findings. While future studies are required to fully explain the driving forces of color associate priming—color diagnosticity, spatial-frequency, and identity specificity have been positively identified as contributing factors and are discussed in detail below.

### Identity Specificity

The color primes appear to have different effects on the object naming and categorization tasks. Though significant interactions between prime color and fruit type were found in the low spatial-frequency conditions of both tasks these two results are highly dissimilar. The follow-up tests in the naming task yielded significant results, but those of the categorization task did not. None of the highly color-diagnostic objects were primed by their diagnostic colors when the participants were required to categorize the fruit objects, which does not provide evidence of color associate priming. This difference between the two tasks appears to be due to the differences in how the participants were required to respond.

In Experiment 2 the participants categorized the objects as being either “fruit” or “car”, but were not required to identify what kind of fruit or car it was. In other research, the ability of an object's color to facilitate its recognition has been shown to be modulated by the level of identity the observer is attempting to determine [40]. Additionally, in the normal time course of object recognition processing the general categories to which an object belongs are determined before its specific identity is achieved [41]–[43]. Therefore, though object recognition normally requires only a fraction of a second, it is highly likely that the participants were responding well before they were able to determine the object's specific identity. In fact, many participants claimed to have used this kind of strategy in the follow-up interviews. Simply put, it seems that the fruit objects were not primed by their color associates in the categorization task simply because the participants were not required to recognize them at such a specific level.

However, the time course of object recognition suggests an alternative interpretation of these results. It may be the case that priming did indeed occur, but it facilitated recognition for objects within the “fruit” category instead of specific fruit types. This would explain the lack of a significant interaction between prime color and fruit type and the highly significant main effect of prime color found for the normal, unfiltered object photographs. As Figure 4 shows, the orange prime produced the fastest reactions across all types of fruit, and the follow-up statistical analyses confirmed that these reaction times were significantly different from most of the other prime colors, which seems to suggest that orange is the color most closely associated with the category “fruit”. This interpretation is particularly intriguing as it could have been predicted based on the evolutionary theories of color vision in primates. In tropical rainforests primates are known to predominantly seek out and consume yellow/orange fruits [3], [5], as these colors are typically indicative of ripeness [7]. Therefore it may be that evolutionary processes have strengthened the link between yellow/orange colors and the recognition of fruits. Alternatively, it may instead be the case that viewing the orange Gaussian color activated the word “orange” which then worked as a semantic prime for the other fruit objects, or it may simply be that the participants had formed strong prior associations between this particular range of fruit types and the color orange. Regardless, the orange prime produced the greatest facilitation for categorizing the fruit objects used in this study, even for types of fruit that do not normally appear orange.

### Spatial-frequency Information

Object recognition is greatly facilitated by the presence of an object's many visual features, including shape, texture and luminance. The facilitatory effects of these features can be so great that the contributions of color information may be diminished to the point of being inconsequential for object recognition purposes [16], [40], [44]. Therefore, in both experiments we attempted to amplify the color priming effects by removing one or more of these visual features from the target photographs.

In Experiment 1 the naming reaction times were measured for the original photographs, photographs that contained only the low spatial-frequency luminance information, and photographs that contained only the high spatial-frequency shape and texture information. All three conditions produced the same general trends, which suggests that the presence or absence of the other visual features did not greatly influence the effectiveness of the color primes. The only obvious difference between the three conditions was in their overall reaction time speeds. The low and high spatial-frequency conditions had much slower reaction times than the normal condition, likely reflecting an increase of task difficulty. Despite this overall similarity, significant color priming effects were only found for the normal and low spatial-frequency conditions; no color priming was found for the high spatial-frequency condition. Interestingly this suggests the color associate priming was driven by the low spatial-frequency luminance information of the target objects. Despite our predictions it does not appear to simply be the case that color will become more informative of object identity when shape information has been degraded. Instead, this result appears to indicate that color information has its greatest influence on the early stages of object recognition, as low spatial-frequencies are known to typically be processed before high spatial-frequencies [48]–[50].

### Color Diagnosticity

Color information is a feature of almost all visual objects. The inclusion of color within an image has been shown to facilitate the recognition of a wide range of objects [10]–[12]. However, studies have shown that the recognition of color-diagnostic objects, objects that are well known to have a specific color [14], generally show the greatest benefit from the inclusion of color information [13], [15], [16]. Therefore, the current study compared the effectiveness of various color primes across a range of objects varying in their color diagnosticity. It was predicted that the most color diagnostic objects (bananas, lemons, oranges, and strawberries) would show the strongest priming effects. It is important to note that the color primes used in this study were not isoluminant, but their luminance differences did not appear to have a measurable influence on the priming effects.

Across the normal and low spatial-frequency conditions of Experiment 1 the bananas, lemons and oranges were found to be differentially influenced by the color primes at a statistically significant level. These objects were most primed by their diagnostic colors (*yellow*-banana, *yellow*-lemon, *orange*-orange), which supports our hypothesis. Though these data may appear somewhat understated, the finding of any such priming is indicative of an interaction between color perception and color knowledge processing. This interaction is likely due to an overlap between the processing of visually presented colors and the color knowledge associated with these highly color-diagnostic fruit objects. This is a novel finding that provides support for the theory of modality grounded object knowledge, which for highly color-diagnostic objects appears to include color processing areas.

## Conclusions

Colorless object images can be primed by the prior presentation of their known color associates. This color associate priming is based entirely on the color information stored within object knowledge. Three factors that have an influence on this color priming have been identified: the color-diagnosticity of the objects, their spatial-frequency information, and the required level of identification specificity. These findings are in line with the previous behavioral research, and provide support for the theory that object knowledge is grounded within modality specific systems [28], [51] by demonstrating an interaction between the processing of color perception and object knowledge.
